# Assessment of extra-coronary peripheral arteriopathy in spontaneous coronary dissection: state of the art in non-invasive imaging techniques and future perspectives

**DOI:** 10.1093/ehjimp/qyad044

**Published:** 2023-12-26

**Authors:** Emmanuel Androulakis, Christos Kourek, Apostolos Vrettos, Nikolaos Kontopodis, Eirini Lioudaki, Maria Prasinou, Andreas Xanthopoulos, Alexios Antonopoulos, Alexandros Briasoulis, Raad Mohiaddin

**Affiliations:** Inherited Cardiac Conditions Department, St George's University Hospital, Blackshaw Rd, London SW17 0QT, UK; Royal Brompton and Harefield Hospitals, Guy's and St Thomas' NHS Foundation Trust, National and Heart Lung Institute, Imperial College London, Sydney St, London SW3 6NP, UK; Department of Cardiology, 417 Army Share Fund Hospital (NIMTS), Athens, Greece; Medical School of Athens, National and Kapodistrian University of Athens, Athens, Greece; Department of Cardiology, Barts Health NHS Trust, London, UK; Vascular Surgery Unit, Department of Cardiothoracic and Vascular Surgery, Medical School, University of Crete, Heraklion, Crete, Greece; Renal Unit, King's College Hospital NHS Foundation Trust, London, UK; Department of Immunology, Royal Free London NHS Trust, London, UK; Department of Cardiology, University of Larisa, Larisa, Greece; Medical School of Athens, National and Kapodistrian University of Athens, Athens, Greece; Medical School of Athens, National and Kapodistrian University of Athens, Athens, Greece; Royal Brompton and Harefield Hospitals, Guy's and St Thomas' NHS Foundation Trust, National and Heart Lung Institute, Imperial College London, Sydney St, London SW3 6NP, UK

**Keywords:** spontaneous coronary artery dissection, extra-coronary peripheral arteriopathy, non-invasive imaging, fibromuscular dysplasia, computed tomography angiography, magnetic resonance angiography

## Abstract

Spontaneous coronary artery dissection (SCAD) has been recognized as an important cause of acute coronary syndrome in women ≤ 50 years old, and up to 43% of pregnancy-associated myocardial infarction. SCAD has a strong association with extra-coronary arteriopathies, including either more common entities such as dissections, intracranial or other aneurysms, and extra-coronary and coronary arterial tortuosity or less common inherited vascular disorders such as Ehlers–Danlos syndrome, Marfan syndrome, and Loeys–Dietz syndrome, leading to the conclusion that systemic arterial disorders may underlie SCAD. Fibromuscular dysplasia is the most common extra-coronary vascular abnormality identified among these patients, also sharing a common genetic variant with SCAD. The American Heart Association, in a scientific statement regarding the management of SCAD, recommends that patients with SCAD should undergo additional evaluation with imaging techniques including either computed tomography angiography (CTA) or magnetic resonance angiography (MRA). MRA has been shown to have sufficient diagnostic accuracy in identifying extra-coronary arterial abnormalities, almost equal to CTA and conventional angiography. The aim of this review is to appraise the most recent important evidence of extra-coronary arteriopathy in the setting of SCAD and to discuss the strengths and weaknesses of various non-invasive imaging methods for screening of extra-coronary arteriopathies in patients with SCAD.

## Graphical Abstract

**Graphical Abstract:**
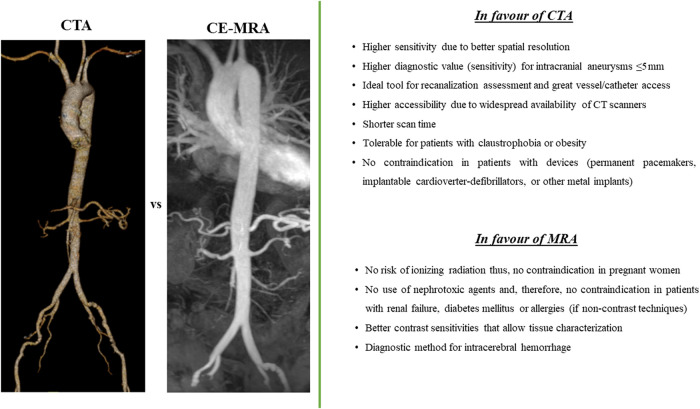

## Introduction

Spontaneous coronary artery dissection (SCAD) has recently been realized as a not so uncommon cause of acute coronary syndrome (ACS) especially among subjects without the typical atherosclerotic phenotype.^1^ Distinguishing SCAD from typical atherosclerotic ACS is important because this is a condition with distinct diagnostic, therapeutic, and prognostic manifestations (*Table 1*).^2–4^ Patients with SCAD are at a higher risk to be misdiagnosed and being discharged after evaluation in the emergency department due to their younger age, female gender, and absence of typical atherosclerotic risk factors.^5,6^ These patients usually present a distinct clinical phenotype that is similar to that of subjects with fibromuscular dysplasia (FMD), with a history of variable clinical symptoms such as hypertension, tinnitus, headaches, and cervical or epigastric bruit.

**Table 1:** Similarities and differences in the diagnostic and therapeutic approach between SCAD and ACS patients

SCAD	ACS
*Clinical presentation*	
Most common symptoms: chest discomfort, chest pain, rapid heartbeat or fluttery	Most common symptoms: acute chest discomfort described as pain, pressure, tightness, heaviness, or burning
Less common symptoms: pain to the arms or neck, nausea or vomiting, unusual or extreme tiredness, shortness of breath, back pain	Less common symptoms: men → shoulder/arm pain, women → diaphoresis, epigastric pain/indigestion, dizziness, nausea/vomiting, jaw/neck pain, shortness of breath
*Diagnostic approach*	
ECG: STEMI alterations (24–87%) Biomarkers: elevation of hs-cTn Echocardiography: reduced LVEF < 50% (44–49% of patients) Other characteristics: complications such as ventricular arrhythmias (3–10%), cardiogenic shock (<3%), and sudden cardiac death (<1%) Imaging techniques: coronary angiography plus OCT or IVUS	ECG: ST-elevation, ST-segment depression, new bundle brunch block Biomarkers: elevation of hs-cTn Echocardiography: ventricular akinesia/hypokinesia and usually impaired LVEF Imaging techniques: coronary angiography is the gold standard
*Therapeutic approach*	
If patient haemodynamically stable, then conservative management including (i) beta-blockers, (ii) aspirin, and (iii) angiotensin converting enzyme inhibitors or angiotensin receptor antagonists or calcium channel blockers or mineralocorticoid receptor antagonists. If patient haemodynamically unstable, then revascularization including PCI or CABG. Antiplatelets and anticoagulants only after stenting. Thrombolysis is contraindicated.	Pharmacological therapy: oxygen, nitrates, opioids, beta-blockers, anticoagulants, antiplatelets. Patients with STEMI → primary PCI or fibrinolysis if primary PCI is not possible within 120 min of diagnosis. Patients with NSTEMI → immediate invasive strategy is recommended if possible. CABG should be considered for patients unsuitable for PCI.
Major differences between SCAD and ACS	
Sex (SCAD most common in females vs. ACS most common in males) Age (SCAD most common in younger patients vs. ACS most common in older patients) Cardiovascular risk factors (patients with SCAD have less cardiovascular risk factors than patients with ACS) Fibromuscular dysplasia (associated with SCAD but not with ACS) Coronary tortuosity (most in SCAD than ACS)	

SCAD, spontaneous coronary artery disease; ACS, acute coronary syndrome; PCI, percutaneous coronary intervention; CABG, coronary artery by-pass grafting; OCT, optical coherence tomography; IVUS, intravascular ultrasound; LVEF, left ventricular ejection fraction; hs-cTnI, high-sensitive cardiac troponin; ECG, electrocardiogram; STEMI, ST-elevation myocardial infraction.

In this regard, the importance of the evaluation of the presence of extra-coronary vascular disease in patients with suspected SCAD by means of non-invasive imaging, including computed tomography angiography (CTA) and magnetic resonance (contrast enhanced) angiography (CE-MRA) has two-folds. First, it can raise the suspicion of SCAD in specific subgroups of patients with ACS, therefore initiating the appropriate diagnostic and therapeutic protocols directed to this entity, and secondly, it may help identify various inter-related clinical conditions from vascular beds other than the coronary circulation. The incidence of extra-coronary disease among SCAD patients has not been completely elucidated. More importantly, the clinical implications of this involvement remain largely unknown. The aim of this document is to revise most recent important evidence and highlight strengths and weaknesses of non-invasive imaging modalities in assessing peripheral vascular pathology in the context of SCAD.

### Epidemiology

In the general population, SCAD is the cause of ACS in 0.2–4% of cases.^7–11^ It is recognized as an important cause of ACS in women and has been reported to account for nearly a quarter of cases of ACS in women ≤ 50 years old,^12,13^ and up to 43% of pregnancy-associated myocardial infarction.^14^ Although classically thought to affect young women, SCAD is now increasingly recognized to also occur in older and post-menopausal women. In a 168 patient-cohort, almost 60% were over age 50, and 62% of women affected were post-menopausal.^15^ SCAD has been reported in all racial backgrounds, but the majority of patients are white, which may represent referral and sampling bias at reporting centres.^15,16^ Although any artery can be affected, the left anterior descending artery is the most commonly affected (in 32–46% of cases) but multivessel SCAD can occur in up to 23% of cases.^8,16,17^ In most cases, the mid to distal segments of coronary arteries are involved.^9^

### Histopathology-pathogenesis

**Figure 1:**
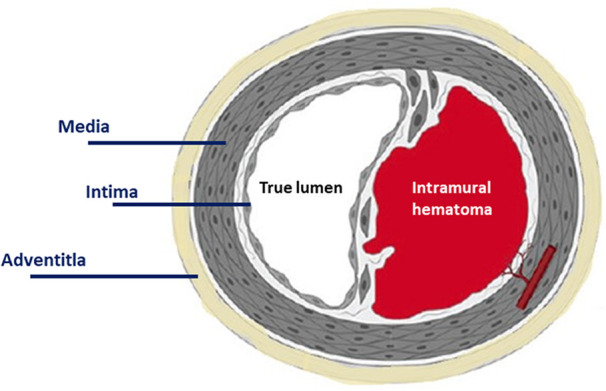
Pathophysiology of spontaneous coronary artery dissection.

SCAD is traditionally defined as the presence of a haematoma within the media of the coronary artery, either directly as the result of intimal tear or due to the creation of intramural haematoma, that leads to luminal encroachment and subsequent myocardial ischaemia and infarction (*Figure 1*). The underlying mechanism of non-atherosclerotic spontaneous coronary artery dissection is not fully understood. An intimal tear or bleeding of vasa vasorum with intra-medial haemorrhage has been proposed,^18^ however, a recent study suggested that the immediate cause of SCAD is likely to be the development of a spontaneous intramural haematoma rather than intimal disruption.^18^ An inflammatory reaction in the adventitia has been also observed, suggestive of peri-arteritis that may potentially breakdown the medial-adventitial layer, and previous histopathological case reports have described SCAD as a mono-arteritis because of the density of the reported associated inflammatory infiltrate.^19^ However, recent evidence suggests that coronary inflammation in SCAD is a localized healing response to the injury rather than a causal vasculitic process.^18^ Connective tissue abnormalities, FMD, postpartum status, multiparity, systemic inflammatory conditions, and hormonal therapy have all been implicated in the pathophysiology of SCAD and are present in up to 80% of SCAD cases.^15^ Precipitating cardio-circulatory stressors are thought to provoke the acute SCAD event on a background of predisposing arteriopathy.

### Role of genetic evaluation

Genetic underpinnings of SCAD are inferred by its occurrence in generally younger patients and in association with minimal cardiovascular risk factors; features that are typical of genetically triggered arteriopathies. A recent study revealed five replicated risk loci and positional candidate genes for SCAD, most of which are associated with extra-coronary arteriopathies.^20^ Although SCAD occurs as a sporadic disorder in most individuals, in a minority of cases, it is associated with known inherited connective tissue and aortopathy syndromes, and although these account for only 5–9% of events, genetic evaluation may be considered in this context.^5^ Genetic testing with a panel of 22 genes associated with familial aortopathies and related connective tissue disorders in a major SCAD registry confirmed the diagnosis of a known inherited connective tissue disorder in 5.1% of the cases, with most cases exhibiting pathogenic mutations in COL3A1 (collagen type III alpha 1 chain).^21^ Non-coronary FMD is detected in up to 86% of SCAD patients, indicating that SCAD may be related to an underlying systemic arteriopathy. In fact, SCAD shares a common genetic variant with FMD.^5^ PHACTR1/EDN1 is a genetic risk locus for several vascular diseases, including FMD and coronary artery disease. In a large genetic study conducted in >1000 SCAD patients and ∼7200 controls, associations were reported between rs9349379, a common non-coding variant in the PHACTR1/EDN1 locus, and the risk of SCAD.^22^ Moreover, rare familial cases composed of two or more affected relatives have been reported, implicating a hereditary predisposition.^23^ Despite the recent advances in SCAD susceptibility gene discoveries, it remains to be determined if they translate into clinically useful genetic testing to predict recurrence or risk in unaffected family members.

### FMD and other systemic arteriopathies

Extra-coronary arterial abnormalities found in the screening of some non-atherosclerotic SCAD cohorts have led to the proposal that a systemic arterial disorder may underlie SCAD. Several arteriopathies have been associated with SCAD, but the reported relative frequency varies widely depending on the patient population, number of imaged vascular beds, and type of imaging used for screening.^15,16,24^ The most common of these arterial abnormalities is FMD, a non-inflammatory non-atherosclerotic systemic arteriopathy, also occurs predominantly in middle-aged women with a variable reporting prevalence between 11% and 86%, presenting with dissection, arterial stenosis, occlusion, and/or aneurysm of medium-sized arteries of the renal, extracranial carotid, and vertebral arterial beds.^15,16^ Although FMD is the most common extra-coronary vascular abnormality identified among patients with SCAD, other arterial abnormalities, including dissections, intracranial or other aneurysms, or extra-coronary and coronary arterial tortuosity, have been observed in patients without imaging signs of FMD.^15,16,24^ The prevalence of intracranial aneurysms in SCAD patients seems to be significantly higher, between 14 and 23%,^15,25^ than the reported prevalence in the general population, and expert consensus documents recommend the systematic screening for brain aneurysm in all patients with history of SCAD.^5^ Isolated cases of SCAD have been reported in association with known inherited vascular disorders, such as Ehlers–Danlos syndrome, Marfan syndrome, and Loeys–Dietz syndrome.^26–28^ However, these instances are rare, and what component represents heritable genetic predisposition to SCAD is yet to be determined. Prevalence of extra-coronary peripheral arteriopathies in patients with SCAD is demonstrated in *Table 2*.^12,13,17,25,29–35^

**Table 2:** Studies demonstrating prevalence of extra-coronary peripheral arteriopathy in spontaneous coronary artery dissection

Study	Year	Country	Number of participants (♂/♀)	Consecutive screening	Imaging techniques	Total number of patients with lesions FMD *n* (%) Intracranial aneurysms *n* (%)	Other significant findings
Toggweiler S. *et al*. (2012)^29^	2003–2009	Switzerland	12 (3/9)	Yes	Whole-body MRA and duplex sonography of the renal and carotid arteries	3 (25%) female patients with abnormalities of the renal arteries 2 (17%) 0 (0%)	1 (8%) patient with spontaneous renal artery dissection Non-significant renal artery stenoses
Prasad M. *et al*. (2015)^25^	2010–2014	USA	115 (6/109)	Yes	SCAD CTA protocol of the neck, chest, abdomen, and pelvis in 95 patients plus outside studies with CTA/MRA in 20 patients	76 (66%) patients with abnormalities of the cervical, thorax, visceral, pelvic, and/or intracranial arteries 52 (45%) 9 (8%)	9 (8%) patients with iliac dilatation 9 (8%) patients with coeliac dilatation 5 (4%) patients with carotid dissection 4 (3%) patients with splenic aneurysm
Bastante T. *et al*. (2015)^30^	2011–2014	Spain	8 (1/7)	Yes	CTA in 4 patients and angiography in 4 patients	1 (12.5%) male and 5 (62.5%) female patients with abnormalities of the renal, iliac, and intracranial arteries and the supra-aortic trunks 5 (62.5%) 1 (12.5%)	1 (12.5%) patient with splenic aneurysm
Rashid HN. *et al*. (2016)^13^	2013–2014	Australia	11 (NA)	No	CTA of their renal, iliofemoral, and carotid arteries	3 (27%) patients with extra-coronary vascular abnormalities 2 (18%) 0 (0%)	1 (9%) patient with splenic artery aneurysm
Nakashima T. *et al*. (2016)^12^	2000–2013	Japan	25 (NA)	No	CTA, MRA, or ultrasonography	6 (24%) patients with extra-coronary vascular abnormalities 5 (20%) NA	1 (4%) patient with carotid dissection
McGrath-Cadell L. *et al*. (2016)^31^	NA	Australia	19 (NA)	No	CTA, MRA, or ultrasonography	9 (47%) patients with extra-coronary vascular abnormalities 7 (37%) NA	2 (10.5%) patients with extra-coronary aneurysms (location not reported)
Rogowski S. *et al*. (2017)^17^	1998–2015	Switzerland	40 (NA)	No	Catheter angiography for femoral ± renal vessels	At least 5 (12.5%) patients with extra-coronary vascular abnormalities 5 (12.5%) NA	NA
Saw J. *et al*. (2017)^32^	2012–2016	Canada	327 (30/297)	Yes	CTA or catheter angiography for renal and iliac arteries and cerebrovasculature	At least 234 (71.6%) patients with extra-coronary vascular abnormalities 205 (62.7%) (renal arteries > iliac arteries > cerebrovasculature) 29 (8.9%)	NA
Macaya F. *et al*. (2018)^33^	2016–2017	Spain	40 (4/36)	Yes	Cervical and abdominopelvic MRA	16 (40%) patients with extra-coronary vascular abnormalities 5 (12.5%) 0 (0%)	2 (5%) patients with non-cranial aneurysms at the coeliac trunk and the splenic artery 6 (15%) patients with arterial tortuosity (renal, carotid, aortic, or vertebral artery) 3 (7.5%) patients with focal stenoses in renal and/or carotid artery
Persu A. *et al*. (2022)^34^	2015–2019	UK	173 (6/167)	No	Head to pelvis MRA in all patients and CTA in 43 patients	At least 60 (36%) patients with extra-coronary vascular abnormalities 55 (30.8%) {renal [27 (15.6%)] > cerebrovascular [23 (13.3%)] > iliac [17 (9.8%)] > visceral [5 (2.9%)] arteries} 3 (1.7%)	13 (7.5%) patients with aneurysms 3 (1.7%) patients with dissections 14 (8.7%) patients with focal stenosis Similar performance of MRA and CTA for detection of extra-coronary arterial abnormalities
Visina J. *et al*. (2022)^35^	NA	USA	193 (11/182)	Yes	Brain to pelvis CTA	At least 97 (50.3%) patients with extra-coronary vascular abnormalities 97 (30.7%) NA	20 (10.4%) patients with non-coronary dissection

MRA, magnetic resonance angiography; FMD, fibromuscular dysplasia; CTA, computed tomography angiography; SCAD, spontaneous coronary artery dissection; NA, not applicable.

It is already known that aortic stiffness may lead to left ventricular hypertrophy and altered coronary perfusion.^36^ Specifically, left ventricular hypertrophy may impair coronary flow reserve through the increase of microvessel resistance in the coronary arteries.^37^ As a result, aortic stiffness could be associated with coronary microvascular dysfunction in patients with or without obstructive coronary artery disease.^36^ It has also been proven that coronary microvascular dysfunction, defined as elevation in the index of microcirculatory resistance and/or limited coronary flow reserve, has been associated with SCAD.^38^ Increased arterial stiffness is associated with endothelial dysfunction and LV diastolic dysfunction.^39,40^ Coronary microcirculation is impaired in patients with essential hypertension.^41–44^ Studies have demonstrated a reduced coronary flow reserve in hypertensive patients or in patients with pre-hypertension compared with healthy controls.^41,42^ The combination of elevated LV diastolic filling pressures induced by increased arterial stiffness and vascular damage affecting the aorta and the coronary arteries may explain the existence of coronary flow reserve in hypertensive patients.^39,40,45,46^ This coronary microcirculatory impairment may occur in very early stages of hypertension, even in the absence of LV hypertrophy.^47,48^ Therefore, increased arterial stiffness could be linked with impaired coronary microcirculation and, therefore, with SCAD, especially in hypertensive women.

SCAD has a strong association with extra-coronary arteriopathies and it is recommended by expert consensus statements (IC) that patients with SCAD undergo additional cross-sectional imaging with either CTA or MRA.^3,6,7,15,24,25,49,50^ It can manifest as arterial stenosis, aneurysm, tortuosity, or dissection of potentially any medium-sized artery (although carotids and renal arteries are most commonly affected). On CT, the most common appearances of FMD are aneurysms formation and beading of the renal or other visceral arteries with alternating areas of stenosis and dilatation.^51^ In the majority of cases, this can be present in multiple areas (multi-focal FMD), but in <10% may appear angiographically as a single concentric or tubular narrowing (focal FMD). Patients with SCAD should be clinically screened in order to rule out systemic/syndromic conditions (*Table 3*).^5^ Other vascular abnormalities such as dissections, aneurysms (including intracranial aneurysms), or extra-coronary and coronary arterial tortuosity have also been identified in patients who do not have a formal diagnosis of FMD.^17,52,53^ It is not clear whether these represent insufficiently investigated FMD cases. The use of a low-dose/high-pitch head to pelvic CT can be utilized to rule out FMD in most vascular beds and protocols combining cardiac and extra-cardiac evaluation have been described and should be considered to minimize radiation and contrast dose.^54^

**Table 3:** Screening for ruling out extra-coronary arteriopathies and connective tissue disorders in patients with SCAD

*Personal medical history*
Early-onset hypertension, stroke, pulsatile tinnitus, migraine headaches, renal infarction, subarachnoid haemorrhage, aneurysm or dissection (aortic, peripheral, brain), rupture of hollow organs (intestinal, bladder, uterine), pneumothorax, tendon or muscle rupture, joint dislocation, umbilical or inguinal hernia, scoliosis or pectus deformity, pregnancy, complications (cervical incompetence, haemorrhage, uterine prolapse, hypertension), poor wound healing, ectopia lentis, myopia, detached retina, early glaucoma, or early cataracts, tall stature, valvulopathies, systemic inflammatory disease
*Family medical history*
Dissection (coronary, aortic, peripheral), inherited arteriopathy or connective tissue disorder, fibromuscular dysplasia, aneurysm (aortic, peripheral, brain), early stroke, early myocardial infarction, sudden cardiac death
*Current condition*
Headaches, pulsatile tinnitus, postprandial abdominal pain, flank pain, claudication, easy bruising, joint hypermobility or laxity
*Diagnostic methods*
Blood sample biomarkers
Imaging techniques (ultrasound, CTA, MRA)

It has been recently indicated that among patients with SCAD, those diagnosed with FMD are significantly older than those without. While an apparent explanation for this finding is not obvious, it may be that patients with an early presentation of SCAD may have not yet developed the angiographic appearance of FMD that can later evolve.^55^ Except for FMD, other vascular lesions are often found in patients with SCAD at a rate of 60–70%. These can be found in both FMD and non-FMD patients, being significantly more common in the former group and regard aneurysms, tortuous and/or ectatic vessels, dissections, focal stenosis, and luminal irregularities.^33^ The most common extra-coronary arteriopathies in patients with SCAD are demonstrated in *Table 4*.^5,33,34,49^

**Table 4:** The most common extra-coronary arteriopathies in patients with SCAD

*FMD lesions (17–86%)*
Most frequent arteries
Renal (79.7%)
Carotid (extracranial and intracranial) (74.3%)
Vertebral (36.6%)
Mesenteric (26.3%)
Less frequent arteries
Iliac (9.8%)
Visceral (2.9%)
*Dissections (1.7–19.7%)*
Most frequent arteries
Carotid (75%)
Renal (21.6%)
Vertebral (17%)
Less frequent arteries (<5%)
Mesenteric, cerebrovascular, coeliac, iliac, visceral, aortic
*Aneurysms (7.5–17%)*
Most frequent arteries
Renal (32.9%)
Carotid (21.1%)
Aortic (19.7%)
Coeliac (15.8%)
Cerebrovascular (11.8%)
Less frequent arteries (<5%)
Visceral, intracranial, mesenteric, basilar, vertebral, subclavian, popliteal, iliac
*Focal stenosis (7.5–8.7%)*
Most frequent arteries
Renal
Cerebrovascular
Coeliac trunk
Less frequent arteries (<5%)
Carotid, visceral, iliac

### Non-invasive diagnosis of SCAD and associated extra-coronary pathology

#### Role of CT imaging

Coronary CT angiography (CCTA) allows the non-invasive visualization of the arterial lumen wall as well as of the arterial wall. It has adequate spatial and temporal resolution for the detection of SCAD in the proximal and middle portions of most coronary vessels but may be limited for the assessment of the more distal coronary territories that are frequently affected.^56^ The most common coronary CCTA features of SCAD are absence of atherosclerotic plaque, presence of dissection flap, long-segment of tapered or abrupt luminal stenosis that is described as a sharp demarcation between contrast-opacified patent coronary artery lumen and distal luminal narrowing, intramural haematoma with haemorrhage within the wall of the coronary artery, and perivascular fat stranding.^57^ Patients with SCAD have a high prevalence of coronary artery tortuosity and myocardial bridging, both of which can easily be identified on CCTA.^52,58^

The absence of dissection on CTCA does not effectively rule out SCAD and the use of a multi-modality imaging approach may be warranted; invasive coronary angiography provide sufficient spatial resolution of smaller, more distal, coronary arteries (particularly of those <1.5 mm), and cardiac MRI enables the assessment of myocardial scar and viability. Although current guidelines do not recommend CTCA as a first-line investigation for acute SCAD, there is an emerging role for the monitoring for spontaneous healing and recanalization particularly of larger calibre proximal coronary arteries. Given the known risk for iatrogenic dissection in acute SCAD, routine follow-up invasive angiography may not be recommended whereas CCTA may have an important role, especially in the presence of recurrent symptoms.^3,8^

Series with angiographic follow-up data have shown that in most cases SCAD heals with restoration of a normal coronary architecture that can be confirmed on CCTA. Atherosclerotic CAD may sometimes be difficult to distinguish from SCAD, and CCTA allows the assessment of the lumen and plaque consistency in those ambiguous cases; the presence of persistent stenosis and coronary calcium or positively remodelled atherosclerotic plaque (which is best seen best with CTCA) can be more suggestive of an atherosclerotic event as opposed to SCAD.^59^ CCTA also is useful in ruling out other mimics of SCAD; coronary vasospasm may cause diffuse or focal luminal stenosis, but concurrent presence of intramural haematoma or perivascular fat stranding would point towards SCAD.^60,61^ Coronary artery embolism usually involves multiple coronary artery territories and demonstrates luminal occlusion adjacent to branch points. The presence of an embolic source also favours the diagnosis of coronary artery embolism. CCTA can also offer prognostic information in SCAD, as the absence of residual dissection at CCTA at 3–6 months has been shown to confer excellent long-term prognosis.^59^

#### Role of MRA in SCAD-related peripheral vascular evaluation

MRA has been shown to have sufficient diagnostic accuracy in this setting.^33^ Specifically, a 96% sensitivity and 93% specificity have been reported for the diagnosis of renal FMD, compared with conventional angiography, especially in the presence of string of beads appearance.^62^ Specific techniques during image acquisition such as real-time contrast bolus monitoring, elliptical centric view ordering, and parallel imaging have been proposed to increase diagnostic accuracy of MRA and counteract the disadvantage of the lower spatial resolution compared with CTA.^6^ MRI from head to pelvis is usually employed to evaluate patients with FMD in order to capture the most commonly affected arterial beds (renal, extracranial carotid, and vertebral arteries).^34^

Previous reports that used MRI to screen patients with SCAD for FMD have indicated a high rate of co-existence of these conditions. Specifically, over 60% of patients with SCAD have been reported to present FMD, with multi-focal involvement being the most common type. Renal arteries were most commonly affected, followed by carotid and vertebral arteries, while extremity arteries like brachial and iliac were involved in a minority of patients.^32,55^ If complete screening is not undertaken, cases with FMD may escape diagnosis and in this case, lower co-prevalence rates have been reported (i.e. the Canadian Registry indicated an overall FMD rate of 31% among the study cohort and 58% among the subgroups of patients with complete screening).^11^

**Figure 2:**
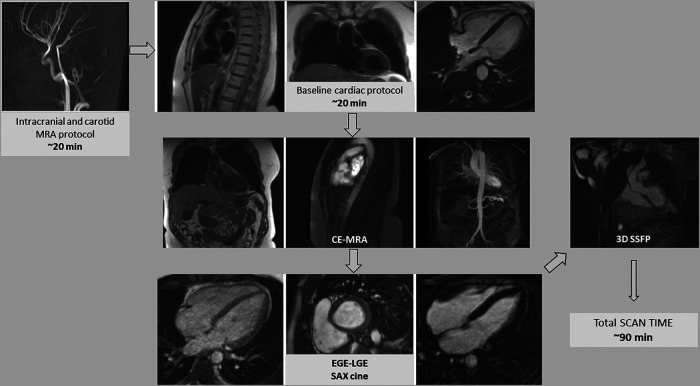
This diagram graphically depicts the timeline of an example study including full vascular screening using the gadobutrol bolus. CE-MRA, contrast-enhanced magnetic resonance angiography, 3D steady-state-free-precession (SSFP); EGE, early gadolinium enhancement; LGE, late gadolinium enhancement; SAX, short axis.

**Figure 3:**
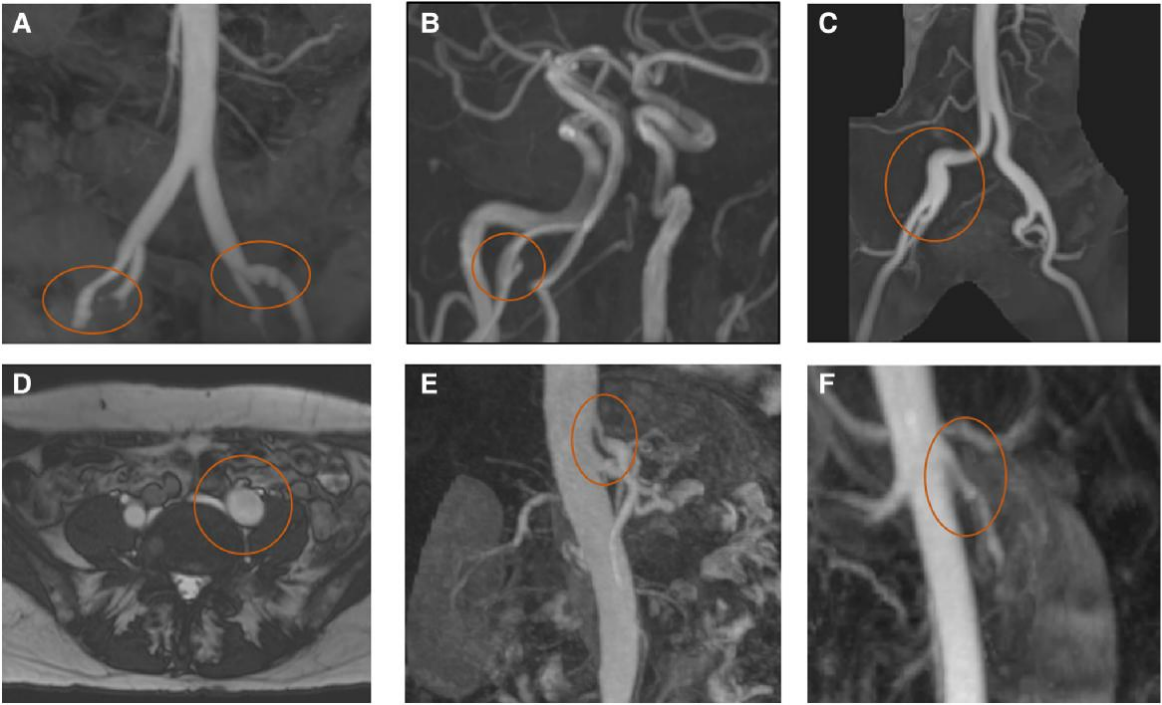
(*A*) Irregularities and focal dilatation in the external iliac arteries. (*B*) Small right vertebral artery focal dissection. (*C*) Tortuous right common iliac artery with mildly dilated middle and distal segments. (*D*) Tortuous distal abdominal aorta and iliac arteries with focal proximal dilation of the left common iliac artery. (*E*) Significant tapering at the origin of coeliac trunk. (*F*) Focal regions of stenosis and mild dilatation in the mid-course of the renal artery typical for fibromuscular dysplasia.

One of the largest cohorts of patients with SCAD screened for peripheral vascular pathology by MRA and to assess SCAD-related infarct size and relevant associations showed CMR is a valuable contribution to the investigation of patients with SCAD. The timeline of an example study including full vascular screening is demonstrated in *Figure 2*. At least 27% demonstrated at least an extra-coronary vascular abnormality; 25% of them were diagnosed with FMD, 30% showed arterial tortuosity, 20% had focal stenoses (not meeting criteria for focal fibromuscular dysplasia as defined by joint European-American consensus criteria)^63^ 20% had ectasia, and 25% exhibited fusiform dilation of proximal aorta. Notably, the majority of patients with positive vascular screening (73.3%) exhibited myocardial infarction (odds ratio, 7.0). In addition, patients with negative vascular screening were more likely to have single-territory involvement as opposed to multiple territories (odds ratio, 4.0).^64^ Different cases of extra-coronary artery abnormalities can be found in *Figure 3*.

**Figure 4:**
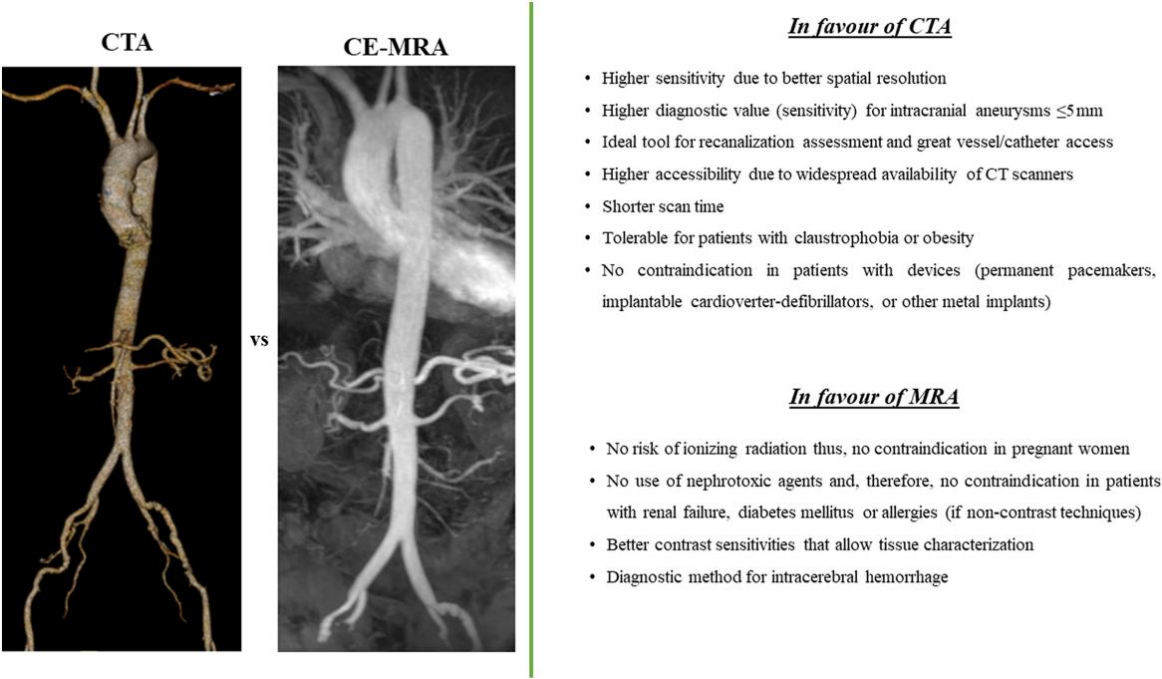
Advantages and disadvantages of each non-invasive imaging technique for identifying extra-coronary arteriopathies in patients with SCAD. (*Left*) Computed tomography angiography (CTA). (*Right*) Contrast-enhanced magnetic resonance angiography (CE-MRA).

**Figure 5:**
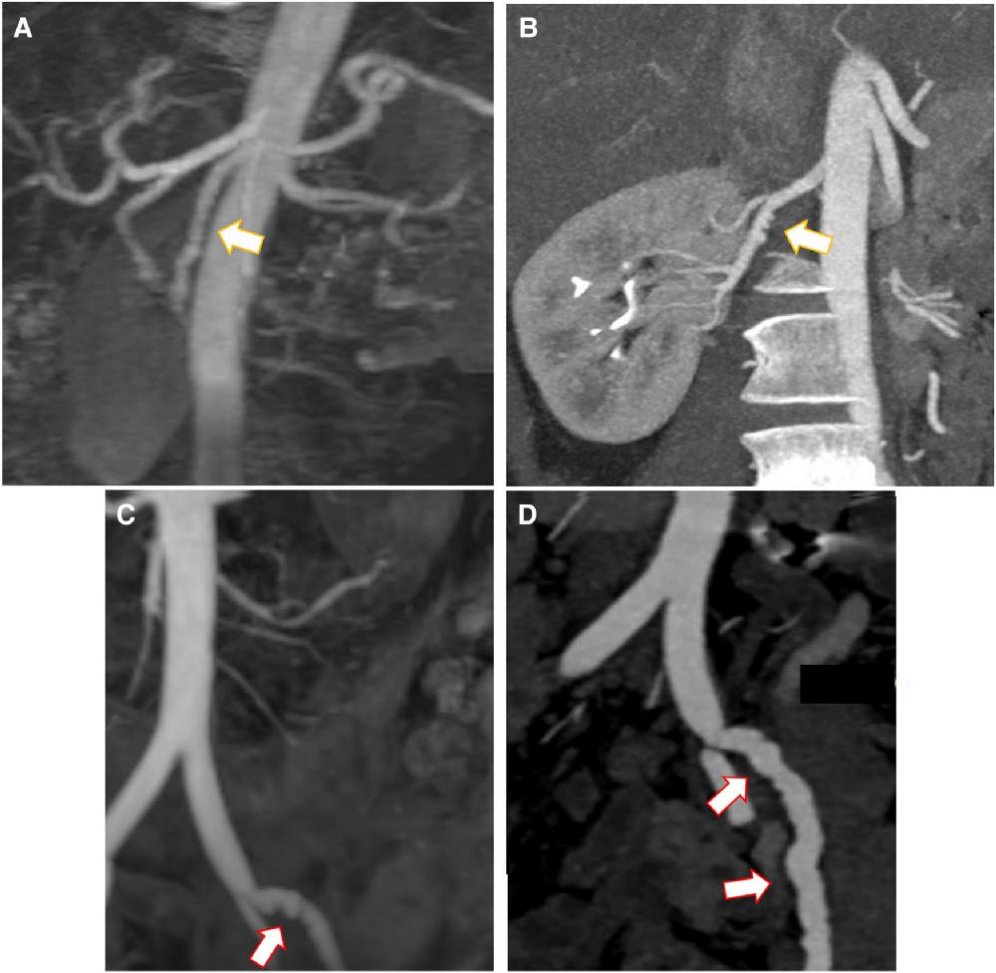
Fibromuscular dysplasia in proximal renal artery; comparison between contrast-enhanced magnetic resonance angiography (*A*) and computed tomography angiography (*B*). Fibromuscular dysplasia in iliac artery; comparison between contrast-enhanced magnetic resonance angiography (*C*) and computed tomography angiography (*D*).

**Figure 6:**
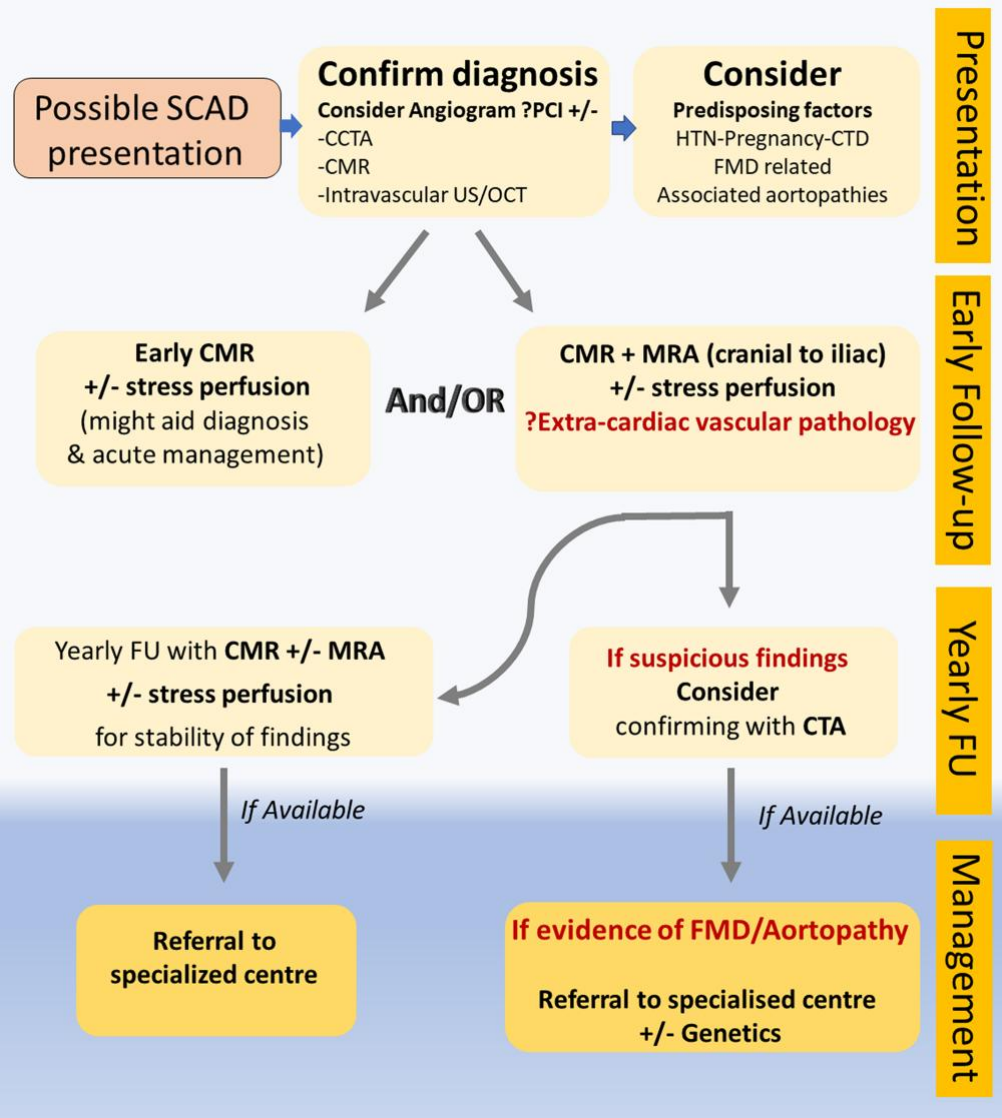
Clinical algorithm for the diagnosis and management of extra-coronary arteriopathies in the setting of SCAD.

Comparison between the two non-invasive diagnostic methods, CTA and MRA, in SCAD findings and extra-coronary arterial abnormalities is presented in *Figures 4* and *5*. CTA seems to have higher accessibility, lower cost and time of examination for the patient, as well as higher diagnostic accuracy in specific cases, but MRA presents less contraindications due to the lack of ionized radiation and nephrotoxic agents (if non-contrast techniques are used), better contrast sensitivities, and lower complexity after image processing. *Figure 6* is demonstrating a clinical algorism for the diagnosis and management of extra-coronary arteriopathies in the setting of SCAD.

### Impact on prognosis based on non-invasive imaging

To which extent the abovementioned common extra-coronary arterial lesions in patients with SCAD have clinical implications is largely unknown. Nevertheless, most previous reports seem to converge in a very low rate of evident vascular clinical events. Indeed, even during a long-term follow-up of 5 years, no primary extra-coronary events were recorded in a series of 173 patients. At the same time, recurrent coronary events were as common as 20% during the same time interval.^55^ Others have indicated similar findings with ∼10–20% rate of recurrent SCAD resulting in major adverse cardiac events during mid-term follow-up, while rate up to 50% using extended follow-up have been reported.^15,65^

Identification of a subgroup of SCAD patients with a higher risk for recurrence would certainly be desirable, but relevant risk factors have not yet been determined. Limited data suggest that severe coronary tortuosity may be a risk factor for recurrent symptomatology, although it is not clear if tortuosity has a direct causal effect for SCAD or if it simply acts as a marker of a most severe vascular involvement and a higher risk patient phenotype.^32,52^ Additionally, hypertension has been identified as a significant predictor of recurrent cardiac events, while use of a beta-blocker seems to confer a significant protective effect.^32^ Notably, the presence of extra-coronary arterial involvement in the form of either FMD or non-FMD has not been found to be related to the risk of SCAD recurrence.^32^ The fact that FMD has been previously related to an increased tortuosity of coronary vessels, which in turn has been found to predispose to adverse cardiac events during follow-up of patients with SCAD, indirectly forms a basis for a hypothetical causal relationship between FMD and SCAD recurrence, but this cannot be supported by available evidence.

### Discussion and future perspectives

Screening for extra-coronary arteriopathies is mainly encouraged by expert consensus statements.^3,5^ As previously mentioned, the impact on prognosis of extra-coronary vascular lesions in subjects with SCAD in patients’ prognosis, as well as the clinical implications for the individual patient, is yet ill determined. One aspect of a possible impact would be the rate of significant extra-coronary vascular events. Available data suggest a very low rate of clinical vascular events and complications in patients with SCAD, which might question the clinical benefit and the cost-effectiveness of screening these patients at all. Another aspect would be the predictive role of extra-coronary vasculopathy in predicting cardiac adverse events and SCAD recurrence. Regarding this, a predictive role of extra-coronary disease in patients with SCAD in terms of risk for recurrence has not been definitively established. The current rationale for extended extra-coronary vascular imaging could be driven by the reported finding of a higher-than-expected proportion of aneurysmatic lesions, with the potential adverse associated risks.^3,5^ However, the benefits of detecting mild extra-coronary abnormalities are uncertain over the potential individual risks in these patients. The potential psychological distress caused by the identification of a mild aneurysm of low risk of future vascular event could lead to additional surveillance imaging with increased radiation exposure of the patient. Moreover, physical and emotional stress is a risk factor of vascular dissection, aneurysm, and cardiovascular event. There is only indirect evidence that may imply causality, but available data cannot yet support such an argument, except in a hypothetical basis. However, we have recently shown important evidence supporting adverse CMR features and larger infarctions in patients with positive extra-coronary vascular screening and multi-territorial coronary involvement.^64^ This may have implications on their long-term follow-up, which may be required for increased cardiovascular risk and events. In our opinion, the fact that except for co-prevalence, a clinical relevance has not been established for FMD or non-FMD arteriopathy in patients with SCAD, may be due to that fact that these patients represent a heterogenous group, and possible causal correlations may be hidden.

Therefore, future research could focus on the special characteristics of subgroups of patients that may be relevant to answer questions such as: is there a possible role of the number of vascular beds involved? Could the form/type of the arterial lesions (such as dissection, focal or multi-focal stenosis, and aneurysms) be relevant when evaluating the effect of extra-coronary vascular disease? Is the symptomatic vs. asymptomatic status of FMD patients relevant when examining possible relations with SCAD? This could identify group of patients who present specific characteristics that may be relevant to define prognosis. The need to establish SCAD registries in big university and non-university hospitals worldwide in order to post and update information regarding baseline characteristics of these patients, risk factors, and clinical characteristics of SCAD lesions is obvious. The development of a global network of SCAD, including all national registries, could provide valuable information and provide new insights in the early diagnosis of this syndrome through the use of non-invasive imaging techniques, as well as future developing biomarkers.

## Conclusions

SCAD presents a strong association with extra-coronary arterial abnormalities, including dissections, intracranial or other aneurysms, or extra-coronary and coronary arterial tortuosity, leading to the conclusion that systemic arterial disorders may underlie SCAD. FMD is the most common extra-coronary vascular abnormality identified among these patients. Other known inherited vascular disorders, such as Ehlers–Danlos syndrome, Marfan syndrome, and Loeys–Dietz syndrome, are rare and observed only in isolated cases. Additional screening of SCAD patients with imaging techniques including either CTA or MRA may be beneficial for their evaluation, as well as the diagnostic and therapeutic approach of an extra-coronary arterial entity. Although non-invasive imaging may not be as sensitive as invasive angiography to detect subtle extra-coronary vascular abnormalities, recent advances in image resolution have given us the possibility to detect most extra-coronary vascular abnormalities with CTA and/or MRA. In fact, CTA provides more reproducible image quality at higher resolution, with easier learning algorithms for the physicians and less cooperation required by patients, compared with MRA. However, specific techniques during image acquisition have been proposed to increase diagnostic accuracy of MRA and counteract its disadvantages compared with CTA. Nevertheless, further clinical trials are required in order to compare various non-invasive imaging techniques in identifying extra-coronary arterial abnormalities and establish appropriate screening, diagnostic and therapeutic protocols of patients with SCAD.

## Data Availability

No new data were generated or analysed in support of this research.

## Lead author biography

Emmanuel Androulakis graduated from University of Athens, and moved to the UK (2012) following his PhD in genetics in hypertension and vascular endothelium. Subspecialized in Multi-Modality CVI with focus on CMR and Inherited Cardiac Conditions/Sports Cardiology. Worked in dedicated clinical and research posts with CMR and ICC teams at Royal Brompton, St George’s, and St Bartholomew’s, for several years. His current research focus is applications of advanced CMR techniques including scar microstructure analysis with biomathematical models, parametric mapping, and AI techniques, in pathology discrimination. Has been elected as one of the EACVI Leaders of Tomorrow for 2022–2024 and currently serves in the Editorial Board of *EHJ-IMP*.
